# Obsessive–Compulsive Personality Symptoms Predict Poorer Response to Gamma Ventral Capsulotomy for Intractable OCD

**DOI:** 10.3389/fpsyt.2019.00936

**Published:** 2020-01-09

**Authors:** Maria Eugênia Copetti, Antonio C. Lopes, Guaraci Requena, Isaac N. S. Johnson, Benjamin D. Greenberg, Georg Noren, Nicole C. R. McLaughlin, Roseli G. Shavitt, Eurípedes C. Miguel, Marcelo C. Batistuzzo, Marcelo Q. Hoexter

**Affiliations:** ^1^ Department & Institute of Psychiatry, Hospital das Clinicas HCFMUSP, Faculdade de Medicina, Universidade de São Paulo, São Paulo, Brazil; ^2^ Yale School of Medicine, New Haven, CT, United States; ^3^ Child Study Center, Yale University, New Haven, CT, United States; ^4^ Department of Psychiatry and Human Behavior, Butler Hospital, Warren Alpert Medical School of Brown University, Providence, RI, United States; ^5^ Center of Neurorestoration and Neurology, Providence VA Medical Center, Providence, RI, United States; ^6^ Department of Neurosurgery, Warren Alpert Medical School of Brown University, Providence, RI, United States

**Keywords:** obsessive–compulsive disorder, obsessive–compulsive personality disorder, personality disorders, gamma ventral capsulotomy, functional neurosurgery

## Abstract

Gamma ventral capsulotomy (GVC) is a radiosurgical procedure which aims to create lesions in the ventral part of the anterior limb of the internal capsule (ALIC). It has been used as a treatment option for patients with intractable obsessive–compulsive disorder (OCD) who do not respond to several first-line treatments attempts. However, changes in personality disorder symptoms after GVC have not been investigated. The aims of this study are to investigate changes in personality disorder symptoms after GVC and to search for baseline personality disorder symptoms that may predict clinical response to GVC. Fourteen treatment-intractable OCD patients who underwent GVC completed the Structured Clinical Interview for DSM-IV Personality Disorders (SCID-II) at baseline and one year after the procedure. Wilcoxon signed-rank test was performed to investigate personality disorder symptom changes before and after surgery. Linear regression models were utilized to predict treatment response, using baseline personality disorder symptoms as independent variables. We did not observe any quantitative changes in personality disorder symptoms after GVC, compared with baseline. Higher severity of obsessive-compulsive personality disorder symptoms at baseline was correlated with worse treatment response after GVC for OCD (β = −0.085, t-value = −2.52, p-value = 0.027). These findings advocate for the safety of the GVC procedure in this specific population of intractable OCD patients, in terms of personality disorder symptom changes. They also highlight the importance of taking into account the severity of obsessive–compulsive personality disorder symptoms when GVC is indicated for intractable OCD patients.

## Introduction

Obsessive–compulsive disorder (OCD) is a chronic disease characterized by the presence of obsessions and/or compulsions that are highly distressing and may severely compromise social and occupational functioning in affected individuals (1). The worldwide prevalence of OCD is approximately 2% of the general population (2). Most patients with OCD benefit at least partially from the currently available first-line treatments: exposure and response prevention (ERP) behavioral therapy and selective serotonin reuptake inhibitors (SSRI) and nonselective (e.g. clomipramine) medications (3).

Around 20% of OCD patients do not respond to first-line treatments (4) and less than 1% of OCD treatment seeking individuals do not respond to multiple interventions and have symptoms so severe and debilitating that they are classified as “intractable” (4, 5). In these cases, Gamma ventral capsulotomy (GVC), a radiosurgical procedure which aims to create lesions in the ventral part of the anterior limb of the internal capsule (ALIC), has been used as a treatment option. Efficacy and safety of this procedure have been described in previous reports (6–9), as well as neuropsychological outcomes (9–11). However, little research is devoted to the study of possible personality changes after GVC or similar neurosurgeries (12). Previous studies have used different methodological approaches to evaluate personality and they obtained controversial results (12). Some studies that used standardized instruments demonstrated stability or improvement in personality domains after OCD neurosurgery (13–16). Other studies that were performed through clinical and behavioral observation have suggested post-surgical exacerbations in personality traits, including increases in temperamental instability, disinhibition, irritability, aggressiveness, apathy and neglect after neurosurgery (9, 16–18). On the other hand, there were no studies that investigated changes in personality symptoms that encompass full diagnostic criteria for personality disorders after neurosurgeries. Given that GVC is an ablative and irreversible procedure, studies that systematically evaluate personality disorder outcomes are highly important.

Using different methodology than previous studies (12, 19–22) that sought to analyze changes in general personality and personality traits, we searched for changes in symptoms of personality disorders which are assessed by the Structured Clinical Interview for DSM-IV Personality Disorders (SCID-II) (23). Assuming that most, if not all, of the SCID-II symptoms are written in such a way as to imply maladjustment (24), in our study, we chose to analyze the scale by considering each symptom as an indicative characteristic of pathology even if the full criteria for the diagnosis of personality disorder was not met.

Therefore, in this study we had three main objectives: First, we aimed to verify the safety of GVC with respect to changes in personality disorder symptoms obtained through the SCID-II. We expected no significant deterioration in personality disorder symptoms after GVC. Second, we aimed to verify whether treatment responders and non-responders differed in terms of personality disorder symptom changes. We hypothesized that there would be no difference between responders and non-responders. Third, we attempted to investigate whether personality disorder symptoms can predict a patient's response to GVC. We expected that the presence of more personality disorder symptoms would predict worse response to GVC, since these symptoms are indicative of psychopathology.

## Materials and Methods

### Sample

Fourteen treatment-intractable OCD patients who underwent GVC treatment from our previous studies (6, 25) completed the SCID-II personality assessment at baseline; 13 completed it one year after radiosurgery and one subject completed the assessment 24 months after the procedure.

The inclusion criteria for intractableness were: a primary diagnosis of OCD according to the DSM-IV (23); a detailed history from previous medical records, clinicians, and family that revealed that the patients had gained minimal or no benefits from previous treatments for OCD; a history of treatment with multiple pharmacologic approaches at maximum doses (e.g., clomipramine hydrochloride, 300 mg/d; fluoxetine hydrochloride, 80 mg/d; paroxetine hydrochloride, 60 mg/d; sertraline hydrochloride, 200 mg/d; and fluvoxamine maleate, 300 mg/d). Participants also had attended at least 20 sessions of cognitive-behavioral therapy (involving exposure and response prevention techniques), without lasting improvement. The appropriateness of their previous pharmacological and cognitive–behavioral therapy regimens was verified by a psychiatrist and by a behavioral therapist.

The study was approved by the Ethical Committee of the University of São Paulo Medical School and all subjects gave written informed consent in accordance with the Declaration of Helsinki.

### Gamma Ventral Capsulotomy (GVC) Procedure

A stereotactic frame was attached to the patient's head and axial and coronal MRI scans were obtained for target localization and dose planning. Targets were defined at the ventral portion of the anterior limb of the internal capsules, 7 to 10 mm rostral to the posterior border of the anterior commissure. Prior to each surgery, the planning procedure was performed jointly by three of the authors (a neurosurgeon and two psychiatrists) and reviewed by two others of the team (a neurosurgeon and a psychiatrist) in close cooperation with the medical physicist.

We used a double-shot technique, in which two distinct isocenters were planned and irradiated on each side of the midline (two in one hemisphere and then two in the other hemisphere, in this sequence). The targets were cross-fired by 201 converging beams of gamma radiation using the 4 mm collimators with a maximum dose of 180 Gy at the center of the targets, corresponding to 100%. The intended volume of necrosis was defined by the 50% isodose line. A Gamma Knife model B (Elekta Instrument AB) was used and thus double bilateral lesions were created (26).

### Psychiatric Evaluation

OCD was diagnosed by two psychiatrists employing the Structured Clinical Interview of the DSM-IV (SCID-I). To assess the severity of obsessive-compulsive symptoms, the Yale-Brown Obsessive Compulsive Scale (Y-BOCS) was used (27). The Beck Depression Inventory (BDI) (28) and The Beck Anxiety Inventory (BAI) (29) were used to assess the severity of anxiety and depression symptoms. The Global Assessment of Functioning (GAF) (30) and Clinical Global Impression (CGI) (31) scales were also utilized to assess overall functioning and improvement. These instruments were administered both prior to and one year after radiosurgery.

Patients were considered responders if they achieved a reduction of at least 35% in their Y-BOCS score and a “very much improved” or “much improved” classification on the CGI scale one year after GVC surgery.

### Personality Disorder Symptom Assessment

Personality disorder symptoms were assessed using the SCID-II instrument, a semi-structured interview based on the diagnostic criteria of personality disorders from the DSM-IV-TR (23). The translated Portuguese version of SCID-II was administered by two trained clinicians (a psychiatrist and a psychologist). The SCID-II is proposed to verify the existence of the symptoms explained in the DSM-IV-TR (23). There is evidence in the literature regarding the consistency between the SCID-II and clinical observation of personality disorder traits (32).

The instrument has 81 questions that are related to different symptoms divided into 10 different personality disorders (as described below). Each of these questions can be independently quantified in terms of the presence of each symptom, ranging from zero (absent), 1 (possible), 2 (probably) to 3 (present).

The diagnosis for each personality disorder requires that at least a minimum amount of specific symptoms are met (sum of “present” symptoms) and can be divided into three clusters, based on similarities. Cluster A includes paranoid, schizoid, and schizotypal personality disorders and their common characteristics are eccentricity. Cluster B includes antisocial, borderline, histrionic and narcissistic personality disorder and their common characteristics are overly dramatic, emotional or erratic behavior. Cluster C includes avoidant, dependent, and obsessive-compulsive personality disorders and their common characteristics are anxiety and fearfulness (23).

In the present study, we used the total number of personality disorder symptoms for each personality disorder as a dimensional score of severity. Patients scoring “probably” or “present” were considered to have that specific symptom. It is important to note that none of the patients had scored “possible” on any personality question. Though there is currently no well-accepted and validated universal classification to determine the severity of personality disorders, several authors have suggested that the sum of symptoms of a specific personality disorder represents a measure of severity (33–35). In a study with OCD and OCPD patients, Pinto and colleagues used the sum of the number of clinically significant DSM-IV OCPD symptoms as a marker of OCPD severity (36).

### Statistical Analysis

Descriptive analysis was performed in order to understand the main features of the sample and to visualize the longitudinal changes in personality measures. Median, minimum and maximum were presented given the small sample size. To compare personality and clinical observations before and after surgery, Wilcoxon signed-rank test was performed. On the other hand, to compare pre-surgery features between responders and non-responders, the Mann–Whitney test was performed.

Still, in order to study the relationships between personality variables and treatment response, the pre/post change in Y-BOCS scores was defined by ΔY-BOCS (initial Y-BOCS minus final Y-BOCS divided by initial Y-BOCS times 100) and three linear regression models were adjusted setting the dependent variable as ΔY-BOCS and setting as independent variables, all pre-surgery personality clusters (i) Cluster A; (ii) Cluster B; and (iii) Cluster C. Stepwise variable selection was applied to select variables for these models. Also, the pre/post variations in other clinical variables—such as BDI, BAI and GAF—were calculated and set as dependent variables for three linear regressions with stepwise variable selection as we did for changes in Y-BOCS.

Effect size is presented as the standardized Z-score for Mann–Whitney and Wilcoxon 0signed-rank tests, and was considered “small” if it is less than 0.1, “medium” if between 0.1 and 0.5, and “large” if greater than 0.5 (37). The significance level for all tests was 0.05. All data analysis was carried out in R software, version 3.5.2 (38).

## Results

### Clinical, Demographic And Personality Disorder Characteristics

Median age of patients (n = 14) at surgery was 34 (min = 24; max = 55) years old, with an equal distribution in terms of gender (male = 7) and median of 12.5 (min = 5; max = 18) years of formal education. The median age at onset of OCD symptoms was 10 (min = 5; max = 22) years old, and the median duration of the disorder was 15.5 (min = 5; max = 40) years. **Table 1** describes pre- and post-GVC median, minimum and maximum of each clinical measure including Y-BOCS, BDI, BAI and GAF, as well as the mean % of reduction, effect size, and the respective p-values. OCD subjects who underwent GVC presented a significant improvement in all clinical measures (Y-BOCS, BDI, BAI and GAF).

**Table 1:** Changes in clinical variables.

	Pre M [min; max]	Post M [min; max]	Mean % of change	ES	Wilcox on p-value
Y-BOCS	32.5 [30; 40]	24.5 [0; 40]	34.9%	0.81	0.025
BDI	22 [11; 46]	19 [1; 35]	28.1%	0.58	0.030
BAI	18.5 [3; 60]	10 [3; 37]	34.7%	0.60	0.024
GAF	32 [18; 43]	47.5 [10; 75]	–38.0%	0.60	0.025

M, median; min, minimum; max, maximum; ES, effect size; Y-BOCS, Yale-Brown Obsessive–Compulsive Scale; BDI, Beck Depression Inventory; BAI, Beck Anxiety Inventory; GAF, Global Assessment of Functioning.

Before the procedure, 10 subjects had at least one diagnosis of personality disorder. After surgery, seven fulfilled criteria for at least one personality disorder. With regard to obsessive–compulsive personality disorder (OCPD), seven patients fulfilled diagnostic criteria before GVC and three patients maintained this diagnosis after GVC. Those four patients who did not fulfill OCPD criteria after surgery (four or more symptoms out of seven) according to the SCID-II prior to the surgery, continued to have a great amount of OCPD symptoms after the procedure.

### Changes in Personality Disorder Symptoms

Our analyses showed that there were no significant changes in the number of personality symptoms after GVC for our entire sample, including both responders and non-responders (**Table 2**).

**Table 2:** Pre-post changes in personality symptoms for all patients, responders and non-responders.

	Group	Pre	Post	Mean differences	ES	p-value
		Median [min; max]	Median [min; max]			
Paranoid	All	1.0 [0; 6]	1.0 [0; 5]	0.29	0.15	0.583
	NR	1.0 [0; 6]	1.5 [0; 5]	0.10	0.04	0.886
	R	0.5 [0; 4]	0.5 [0; 1]	0.75	0.27	0.586
Schizoid	All	1.0 [0; 5]	1.0 [0; 5]	0.21	0.07	0.803
	NR	1.5 [0; 4]	1.0 [0; 5]	0.10	0.07	0.824
	R	0.0 [0; 5]	1.0 [0; 1]	0.50	0.00	1.000
Schizotypal	All	1.0 [0; 4]	0.0 [0; 2]	0.50	0.33	0.212
	NR	1.0 [0; 4]	0.5 [0; 2]	0.40	0.17	0.590
	R	1.0 [0; 1]	0.0 [0; 0]	0.75	0.72	0.149
Antisocial	All	0.0 [0; 1]	0.0 [0; 0]	0.14	0.25	0.346
	NR	0.0 [0; 0]	0.0 [0; 0]	0.00	0.00	1.000
	R	0.5 [0; 1]	0.0 [0; 0]	0.50	0.47	0.346
Borderline	All	0.0 [0; 6]	0.0 [0; 3]	0.23	0.15	0.586
	NR	0.0 [0; 2]	0.0 [0; 3]	0.00	0.00	1.000
	R	0.0 [0; 6]	0.0 [0; 3]	1.00	0.00	1.000
Histrionic	All	0.0 [0; 5]	0.0 [0; 5]	−0.46	0.26	0.347
	NR	0.0 [0; 5]	0.0 [0; 2]	−0.22	0.06	0.854
	R	1.5 [0; 3]	2.5 [0; 5]	−1.00	0.40	0.423
Narcissistic	All	0.0 [0; 4]	0.0 [0; 7]	0.00	0.04	0.892
	NR	0.0 [0; 4]	0.0 [0; 2]	0.40	0.35	0.265
	R	0.5 [0; 3]	0.5 [0; 7]	−1.00	0.00	1.000
Avoidant	All	3.0 [0; 7]	3.0 [0; 7]	0.38	0.26	0.353
	NR	3.0 [2; 7]	4.0 [0; 7]	0.11	0.09	0.792
	R	2.5 [0; 6]	2.0 [0; 3]	1.00	0.47	0.344
Dependent	All	3.5 [0; 6]	2.0 [0; 5]	1.07	0.42	0.117
	NR	4.0 [0; 6]	2.5 [0; 5]	0.60	0.24	0.438
	R	3.0 [0; 6]	0.5 [0; 2]	2.25	0.67	0.668
Obsessive– Compulsive	All	4.0 [0; 7]	2.5 [0; 7]	0.50	0.35	0.194
	NR	4.0 [1; 7]	3.0 [0; 7]	0.50	0.32	0.305
	R	2.0 [0; 5]	1.5 [1; 3]	0.50	0.28	0.577

NR, Non Responders; R, Responders; ES, Effect Size.

**Figure 1:**
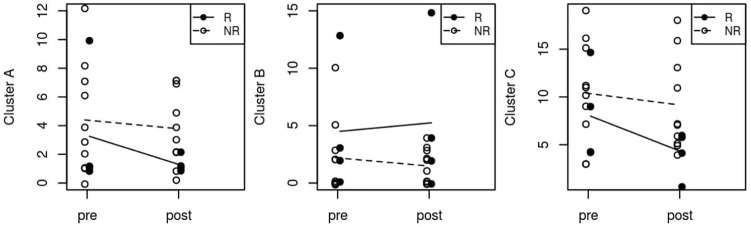
Median pre- and post-surgery personality symptoms in Clusters A, B and C. R, Responder; NR, Non Responder.

Considering the total number of symptoms within clusters A, B and C, we also did not observe any significant change after GVC (**Figure 1**).

### Predictors of Treatment Response

**Figure 2:**
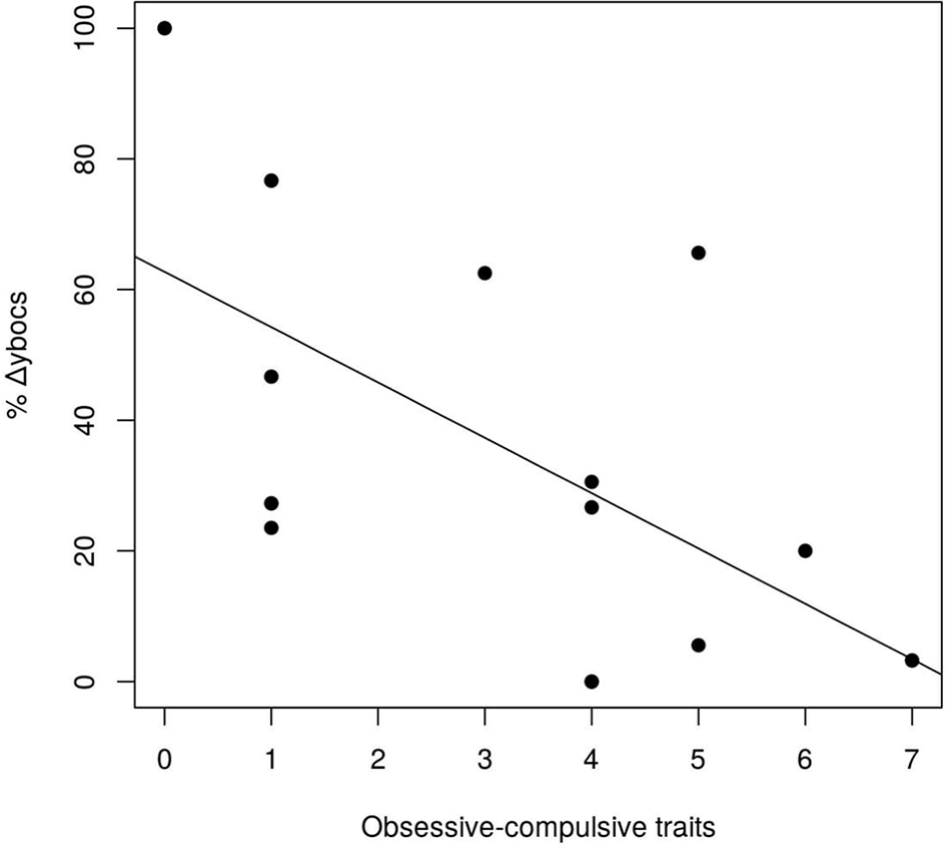
Linear relationship between % of changes in Y-BOCS scores and OCPD symptoms.

Among all the personality disorders evaluated in this study, the number of OCPD symptoms was the only one that was associated with changes in Y-BOCS scores after GVC. Using a step-wise variable selection that included the symptoms of the three personality disorders from Cluster C (avoidant dependent, and OCPD), avoidant and dependent symptoms did not survive in the analysis. Only OCPD symptoms remained in the model (β = −0.085, t-value = −2.52, p-value = 0.027). Thus, the more obsessive–compulsive personality symptoms that a subject possessed before surgery, the worse his/her response was to the procedure, in terms of a reduction in OCD symptoms (**Figure 2**).

We did not find any other associations between any amount of baseline personality symptoms and changes in BAI, BDI and GAF scores, with the exception of borderline symptoms which were associated with changes in BDI scores (β = 0.207, t-value = 3.09, p-value = 0.011).

Finally, given that the main difference in treatment response appears to be driven by OCPD versus non-OCPD status, we also compared patients who met full criteria for the diagnosis of OCPD (n = 5) with the non-OCPD patients (n = 9) in regards to baseline characteristics and treatment response (responders versus non-responders). In all of these analyses, we did not observe any significant difference between-groups (**Table 3**).

**Table 3:** Possible baseline differences between OCD patients with OCPD versus OCD patients without OCPD.

	OCPD	non-CPD
**Age of onset (years-medium)**	**12**	**10**
Wilcoxon test	W = 20.5, p-value = 0.654	
**Duration of OCD (years-medium)**	**13**	**24**
Wilcoxon test	W = 29.5, p-value = 0.564	
**SRI antidepressant (number-medium)**	**5**	**6**
Wilcoxon test	W = 24, p-value = 1	
**Other medications (number-medium)**	**14**	**14**
Wilcoxon test	W = 27, p-value = 0.7947	
**Age at surgery (years-medium)**	**34**	**36**
Wilcoxon test	W = 32, p-value = 0.369	
**YBOCS baseline (score-medium)**	**35**	**32**
Wilcoxon test	W = 15, p-value = 0.2449	
**BDI baseline (score-medium)**	**30**	**16**
Wilcoxon test	W = 12, p-value = 0.124	
**BAI baseline (score-medium)**	**27**	**17**
Wilcoxon test	W = 15, p-value = 0.2496	
**GAF baseline (score-medium)**	**32**	**32**
Wilcoxon test	W = 25.5, p-value = 1	

OCD, Obsessive–compulsive disorder; OCPD, OCD patients with obsessive–compulsive personality disorder; non-OCPD, OCD patients without obsessive-compulsive personality disorder; SRI, Serotonin reuptake inhibitor antidepressant; YBOCS, Yale-Brown Obsessive-Compulsive Scale; BDI, Beck Depression Inventory; BAI, Beck Anxiety Inventory; GAF, Global Assessment of Functioning.

## Discussion

Our study has three main findings. First, it demonstrates the relative safety of GVC for patients with intractable OCD, given that no significant change in personality disorder symptoms was found one year after the procedure. These results corroborate the hypothesis that there was no worsening of personality disorder symptoms in intractable OCD patients after receiving GVC, which suggests the safety of the procedure. The findings are in agreement with Paiva etal. (12) who did not demonstrate any deleterious personality trait changes one year after GVC. Second, we also did not find differences in personality disorder symptom changes in responders versus non-responders. This suggests that the improvement in OCD symptoms is not related to changes in personality disorder symptoms. Third, more OCPD symptoms before surgery predicted worse response to GVC.

It is important to note that other studies as Paiva etal, (12) that focused on assessing personality traits in OCD patients, demonstrated no significant personality changes after GVC. However, in that previous study personality traits were obtained from the Neo Personality Inventory (NEO) (39) and from the Cloninger's Temperament and Character Inventory (TCI) (40) which provide a sense of some basic traits that are central to the description of general personality. Unlike the SCID-II scale (23) used in the current study, the NEO and TCI scales do not intend to measure the pathological functioning of personality and, in these scales, the presence of personality traits is not necessarily related to impairment (41, 42).

Some studies have shown that OCPD is one of the most frequent personality disorders in OCD patients (43–45). The DSM IV defines OCPD as an enduring pattern that leads to clinically significant distress or functional impairment, marked by four or more of the following: i) preoccupation with details; ii) perfectionism; iii) inflexibility about morality and ethics; iv) excessive devotion to work; v) inability to discard worn-out or worthless items; vi) reluctance to delegate tasks; vii) miserliness; and viii) rigidity and stubbornness (23). A study examining the comorbidity of OCPD and OCD found that OCD patients with co-morbid OCPD had a significantly earlier age at onset of initial OC symptoms, earlier age at onset of OCD, more obsessions and compulsions than pure obsessions and showed more impairment in global functioning (46). Other research has shown that patients with OCD and OCPD have significantly greater OCD severity, are more likely to have other psychiatric comorbidities, greater functional impairment, and poorer insight, and suggest that OCPD represents a marker of severity in OCD patients (47).

The worse response of patients with OCPD symptoms to GVC surgery corroborates the findings by Sadri etal. (48) who, in their study, suggest that OCPD may negatively interfere with the efficacy of OCD treatment, especially when treated with Cognitive Behavioral Therapy (48). Our results also resemble findings that show that the presence of OCPD predicted poorer response to pharmacological treatment in OCD (49). Therefore, our results corroborate previous treatment findings and suggest that the presence OCPD symptoms is a predictor of worse response independently of the treatment modality.

Though the relationship between OCPD and OCD has been explored extensively (46, 50, 51), there is an open debate in the literature as to whether or not OCPD may constitute a specific subtype of OCD. Some reports have suggested that patients with both OCD and OCPD have distinct clinical characteristics in several domains compared with OCD patients without OCPD, including more impairment in global functioning, a higher rate of comorbidity, earlier age at onset of initial OC symptoms, and earlier age at onset of OCD (46, 51). Of note, all of these characteristics have been associated with worse treatment response [do (48, 52)]. This opens the door for investigation of different and more targeted interventions for patients who have this comorbidity.

Given that OCPD symptoms may be related to other measures of personality traits, such as neuroticism and flexibility (12, 19), we also performed some additional analyses using data previously collected by our group (10, 12) in order to investigate if OCPD symptoms would be associated with neuroticism (as a dimension from the NEO Personality Inventory) and mental flexibility (evaluated by the Wisconsin Card Sorting Test). These analyses did not show any significant association. We have also shown in another investigation that refractory OCD patients have more frontal lobe symptoms, such as apathy, disinhibition and executive dysfunction when compared with controls (53). In future studies, these measures could also be used to investigate predictors of neurosurgery response. It is important to highlight some limitations of our study. First, the sample size was small, which makes data generalization more difficult. The exploratory nature of the study enables a risk of false positive results due to multiple comparisons. However, it is important to consider that this study was performed with a very specific and rare population, and because it involves a complex procedure with risks, it has very strict inclusion criteria, which makes it difficult to recruit larger samples. In the future, other centres that perform this type of surgery could produce similar analyses with larger samples, in an attempt to corroborate our findings. Second, there is significant difficulty in quantitatively measuring personality symptoms, considering their subjective nature and vast heterogeneity between patients. In addition, although studies have already used the amount of traits as a marker of severity, there is no consensus in the literature in this regard. We suggest that future studies investigate more dimensional forms of personality disorder diagnoses, that include the severity of the pathology. Finally, personality is a stable construct and one-year follow up may not be enough time to find variations. Longer longitudinal studies are necessary to investigate long-term changes in personality symptoms and to verify the safety of this procedure in the long term.

In conclusion, neurosurgical procedures always involve risks and possible side effects, so the verification of safety and the identification of reliable predictors of post-operative outcomes are of great clinical importance. This could lead to more accurate selection of suitable patients, avoiding unnecessary surgeries and improving the overall response rate. Future studies with longer follow-up and larger samples are necessary to confirm and generalize our findings. Additional studies regarding neurosurgical techniques for the treatment of intractable OCD would be valuable in order to confirm the safety of these procedures and to find more characteristics that predict response. Regarding personality disorder symptoms, our analyses showed that there were no significant changes after GVC for the entire sample. This suggests the safety of the GVC procedure in this specific population of intractable OCD patients, during a one year follow-up period. In addition, OCD subjects with OCPD symptoms had a worse response to GVC, suggesting that the presence of OCPD symptoms should be taken into account when GVC is indicated for intractable OCD patients.

## Data Availability Statement

The datasets generated for this study are available on request to the corresponding authors.

## Ethics Statement

The study was approved by the Ethical Committee of the University of São Paulo Medical School (6, 26) and all subjects gave written informed consent in accordance with the Declaration of Helsinki.

## Author Contributions

MC was involved in the implementation of the study, organized the database, and wrote the manuscript. MH, MB, IJ, and AL wrote sections of the manuscript. AL, RS and EM were involved in the design of the study. BG, GN, and NM reviewed the manuscript. GR performed the statistical analysis. MH supervised all steps. All authors contributed to the conceptual planning and discussions and have approved the final manuscript.

## Funding

The work presented here was supported by the National Institute of Developmental Psychiatry (INPD) with grants from Brazilian government agencies FAPESP (#Fapesp 2014/50917-0) and CNPq (#CNPq 465550/2014-2).

## Conflict of Interest

The authors declare that the research was conducted in the absence of any commercial or financial relationships that could be construed as a potential conflict of interest.
